# Chinese version of the international positive and negative affect schedule short form: factor structure and measurement invariance

**DOI:** 10.1186/s12955-020-01526-6

**Published:** 2020-08-24

**Authors:** Jing-Dong Liu, Ri-Hong You, Hao Liu, Pak-Kwong Chung

**Affiliations:** 1 Department of Physical Education, Sun Yat-Sen University, Guangzhou, China; 2 Research Center of Brain and Cognitive Neuroscience, Liaoning Normal University, Dalian, China; 3 Department of Physical Education, Shenzhen University, Shenzhen, China; 4 Department of Sport and Physical Education, Hong Kong Baptist University, AAB 937, Academic and Administration Building, Baptist University Road Campus, Hong Kong, China

**Keywords:** PANAS, Factor structure, Measurement invariance, Validity • reliability

## Abstract

**Background:**

This study translated the International Positive and Negative Affect Schedule Short Form (I-PANAS-SF) into Chinese and examined its factor structure and measurement invariance in Chinese adolescents in Hong Kong.

**Methods:**

A sample of 4136 Chinese adolescents in Hong Kong was invited to complete a set of questionnaires. The factor structure of the I-PANA-SF was examined using confirmatory factor analysis (CFA) and exploratory structural equational modeling (ESEM). Internal consistency reliability was evaluated using Cronbach’s alpha coefficients, and nomological validity was assessed using bivariate correlations between positive affect (PA) and negative affect (NA) subscales with effort, worry and lack of concentration. Finally, measurement invariance across genders and grades was examined to evaluate the invariance of the I-PANAS-SF.

**Results:**

Factor structure analysis suggested that the ESEM model outperformed the CFA model. The results of ESEM analysis indicated that one item (“alert”) was problematic and a 9-item two-factor measurement model with that item removed was a better fit for the data. The Cronbach’s alpha values were above 0.70 (0.81 and 0.83), revealing excellent internal consistency reliability. The PA subscale was positively associated with effort and negatively associated with worry and lack of concentration. The NA subscale was negatively associated effort and positively associated with lack of concentration and worry, indicating nomological validity. Finally, measurement invariance analysis revealed strict invariance across genders and grades.

**Conclusions:**

The results of the study provide preliminary support for validity and reliability of the 9-item Chinese version of the I-PANAS-SF and suggest that it is suitable for use among Chinese adolescents in Hong Kong.

## Background

Affect has drawn extensive attention from researchers in emotion studies and been widely studied in various fields. Watson and colleagues argued that affect could be best represented by two separable but related aspects, the positive affect (PA) and the negative affect (NA), which has been the most widely held view in affect studies [1, 2]. PA reflects the extent to which a person feels enthusiastic, active and alert, whereas NA refers to a state of subjective distress and unpleasurable engagement represented by a variety of aversive feels such as anger, fear, guilt and nervousness [2]. Measurement of affect has been one of the heated topics in affect research. The most widely used instrument assessing PA and NA is the Positive Affect and Negative Affect Schedule (PANAS) [2], which was developed based on previous emotional measurement studies [3, 4]. The PANAS has been proven having good reliability and validity in many studies [2, 5, 6]. It has been translated into different languages [7–10] and modified for different research purposes, including the Positive and Negative Affect Scale for Children (PANAS-C) [11], the Positive and Negative Affect Schedule for Children-Short Form (PANAS-C-SF) [12] and the International Positive and Negative Affect Schedule Short Form (I-PANAS-SF) [13], which were developed based on Watson and colleagues’ fundamental work.

Thompson recently argued that the PANAS has at least two drawbacks, especially in cross-cultural studies [13]. First, it was developed in the United States and some of the words included in the PANAS are either colloquial to North America or are ambiguous in “international” English [6], which may result in different interpretations from respondents from different regions or countries even though English is their native language. Second, many studies of positive and negative affect include numerous other variables in addition to the full 20-item PANAS, which may result in respondents experiencing negative affect in the process of answering the lengthy questionnaires. To address these issues, Thompson developed the 10-item International Positive and Negative Affect Schedule Short Form (I-PANAS-SF) in English with two 5-item subscales measuring PA and NA based on the 20-item PANAS [13]. The I-PANAS-SF is designed to be suitable for use with competent but not necessarily native English speakers and excludes redundant and ambiguous items [13]. The I-PANAS-SF has also been widely used in previous studies and has demonstrated good validity and reliability [13–17]. However, Karim and colleagues found only partial factorial invariance of the I-PANAS-SF among French and Pakistani university students for four items (“active,” “afraid,” “nervous,” and “upset”) [18]. This finding challenges Thompson’s assumption and suggests that translated versions of the PANAS measures are needed. To the best of our knowledge, the I-PANAS-SF has not previously been translated into Chinese.

Although the two-factor structure of affect (PA and NA) has been generally accepted [1, 19], whether or not PA and NA are in fact orthogonal or correlated dimensions has been, and remains, controversial [13, 20, 21]. For the PANAS measure, it was proposed that PA and NA were orthogonal traits and items loaded on either PA or NA with little to no cross-loading on their unintended factor [2]. Previous research has revealed the independence of the PA and NA factors and found negative low-to-moderate inter-factor correlations (− 0.11 to − 0.35) between PA and NA, even when covariance between some item-level errors was taken into consideration [2, 8, 11, 22–27]. This suggested that the two-factor structure of the PANAS was replicated and further implied that there was some overlapping content and some PA and NA items were redundant. Therefore, a two-factor I-PANAS-SF was developed by removing some redundant items from Watson’s PANAS [13]. The two-factor structure of the I-PANAS-SF was also replicated in a previous study and low-to-moderate inter-factor correlations (− 0.18 to − 0.36) were reported [18]. Most previous research has examined the factor structure of the PANAS measures using confirmatory factor analysis (CFA), which has been criticized for relying on a highly restrictive independent cluster model, in which cross-loadings of items on unintended factors in multidimensional instruments are forced to be zero [28]. One proposed method for overcoming the limitations of CFA is exploratory structural equation modeling (ESEM), which integrates the principles of exploratory factor analysis (EFA) within a CFA/SEM framework and provides a better representation of an instrument’s complex multidimensional structures [28]. ESEM has been widely used in studies that aimed to examine the factor structure and measurement invariance of instruments in various domains [29] and could therefore be a suitable approach to exploring the inter-factor correlation between PA and NA.

To facilitate future research on PA and NA in Chinese populations, the purpose of the current study was to translate the I-PANAS-SF into Chinese and further evaluate its factor structure and measurement invariance in a sample of Chinese adolescents in Hong Kong. First, the factor structure of the Chinese translated version of the I-PANAS-SF was evaluated using CFA and ESEM. Next, the internal consistency reliability and nomological validity of the I-PANAS-SF was evaluated. Finally, the measurement invariance of the I-PANAS-SF measurement model was examined across genders and grades.

## Methods

### Participants

A total of 4136 Chinese students (Grades 7–11) from 59 government and government-aided secondary schools in Hong Kong were invited to participate in this study, which measured their feelings about their physical education (PE) classes. By excluding the incomplete data, data from 4111 students were identified as valid for analysis. The participants were aged between 11 and 19 years (M = 14.12, SD = 1.50). All of them could read and speak Chinese. Table 1 shows the demographic statistics of the participants.

**Table 1:** Demographic statistics of participants

Variables	4111
Age (years)	14.24 ± 1.50
Gender	
Male	2002 (48.7%)
Female	2104 (51.2%)
Missing	5 (0.1%)
Grade	
7th	866 (21.1%)
8th	1034 (25.2%)
9th	1204 (29.2%)
10th	587 (14.3%)
11th	420 (10.2%)

### Procedures

Ethical approval was obtained from a local university’s Human and Animal Research Ethics Committee. PE teachers were contacted to obtain permission for data collection. Written informed consent was received from the students and their parents prior to data collection, and detailed information regarding the study was provided. The participants were informed prior to data collection that the anonymity and confidentiality of their answers would be preserved at all times. Participation in the study was voluntary and the administrator of the data collection emphasized that the purpose of the questionnaire was to measure participants’ general feelings about their PE classes. The participants completed the questionnaires at the end of a PE class in the absence of the PE teachers.

### Measures

#### I-PANA-SF

The 10-item I-PANAS-SF includes five items measuring PA and five items measuring NA [13]. Responses were provided on a 5-point Likert scale ranging from 1 (never) to 5 (always). The items were translated from English to Chinese using translation and back-translation techniques [30]. The items were translated independently from English to Chinese by two bilingual translators. Consensus was reached through discussion to form a preliminary Chinese version, which was then independently translated from Chinese back to English by two other translators. Comparison of the back-translated English version with the original English version revealed that the meaning of the items was identical. Finally, 10 native Chinese secondary school students in Hong Kong were invited to complete the Chinese version of the I-PANAS-SF. The students reported that the instructions and items were easy to understand.

#### Lack of concentration

Four modified items from the Sport Anxiety Scale-2 (SAS-2) [31] were used to measure students’ concentration disruption in PE class. For example, “In PE, it is hard for me to focus on what I am supposed to do.” Reponses were provided on a 4-point Likert scale ranging from 1 (not at all) to 4 (very much). The scale demonstrated good internal consistency reliability in a previous study [32].

#### Worry

Four modified items from the SAS-2 [31] were used to measure students’ worry in PE class. For example, “In PE, I worry that I will perform badly.” Reponses were provided on a 4-point Likert scale ranging from 1 (not at all) to 4 (very much). The scale demonstrated good internal consistency reliability in a previous study [32].

#### Effort

Three modified items from the Intrinsic Motivational Inventory (IMI) [33] were used to measure students’ effort in PE class. For example, “I tried very hard in my PE class.” Reponses were provided on a 7-point Likert scale ranging from 1 (strongly disagree) to 7 (strongly agree). The scale demonstrated a good internal consistency reliability in a previous study [32].

### Data analysis

Analyses were conducted using Mplus (Version 7.31). First, the 10-item two-factor measurement model of the I-PANAS-SF was examined using CFA with robust maximum likelihood (MLR). Second, ESEM was conducted using an MLR estimator and an oblique geomin rotation with an epsilon value of 0.5 to reexamine the factor structure and the performance of the items. The fits of the models using CFA and ESEM approaches were compared and the performances of the items were evaluated. Third, Cronbach’s alpha coefficients were calculated to evaluate the internal consistency reliability of the I-PANAS-SF subscales. Forth, the nomological validity of the I-PANAS-SF subscales was evaluated using bivariate correlations of PA and NA with lack of concentration, worry, and effort. Finally, measurement invariance of the scale across genders and grades was investigated using multiple-group ESEM. Four models were evaluated: configural (M1), metric invariance (M2: weak invariance), scalar invariance (M3: strong invariance), and item uniqueness invariance (M4: strict invariance) [34].

Multi-fit indices were used to evaluate the adequacy of the model fit to the data, including the chi-square value, comparative fit index (CFI), Tucker-Lewis index (TLI), root mean square error of approximation (RMSEA) and its 90% confidence interval (CI), and standardized root mean square residual (SRMR). As no specific model-data fit recommendations are available for the ESEM, the commonly recommended criteria for independent cluster model CFA [29] were adopted for ESEM analysis. Acceptable fit thresholds of >.90 for the CFI and TLI, close to (or less than) .08 for SRMR and RMSEA indices were applied. CFI and TLI values exceeding .95, and SRMR and RMSEA close to (or less than) .08 and .06, respectively, represent a good fit [35].

As the chi-square difference test depends on the sample size, the differences in the descriptive fit indices (ΔCFI, ΔRMSEA, and ΔSRMR) were used in this study. According to Chen [36], when testing for metric invariance, a change of ≥.010 in the CFI, supplemented by a change of ≥.015 in the RMSEA or a change of ≥.030 in the SRMR, indicates noninvariance; for testing scale or item uniqueness invariance, a change of ≥.010 in the CFI, supplemented by a change of ≥.015 in the RMSEA or a change of ≥.010 in the SRMR indicates noninvariance. Information criteria, such as Akaike information criterion (AIC), the Bayesian information criterion (BIC), and the sample size adjusted BIC (ABIC) were also used for model comparison. A model with lower values for information criteria was considered to fit the data better than a model with higher values.

## Results

### Factorial structure, descriptive statistics, and reliability

Descriptive statistics for the I-PANAS-SF items are presented in Table 2 and model fit indexes for the different models are presented in Table 3. The 10-item CFA solution demonstrated a poor model fit to the data. The correlation between PA and NA was not significant (r = − 0.01, *p* = 0.72). The factor loading of one item (“alert”) was lower than 0.30 (*λ =* 0.22), which means that the item performed poorly on its intended factor (PA). Examination of the modification index (MI = 834.38) revealed that removal of the item much improved the model. After removing the item “alert”, the 9-item CFA solution demonstrated a marginal model fit to the data (see Table 3). All items were found to be loaded on their intended factors with an average factor loading of 0.63 (ranging from 0.58 to 0.80). The correlation between PA and NA was not significant (r = − 0.04, *p* = 0.09).

**Table 2:** Summary of the item means (M), standard deviation (SD), factor loadings, and Cronbach’s alpha coefficients (α)

I-PANAS-SF			10-item CFA		9-item CFA		10-item ESEM			9-item ESEM		Alpha
	M	SD	NA	PA	NA	PA	NA	PA	alpha	NA	PA	
NA subscale									0.832			0.832
1 Upset	1.89	1.120	0.784	–	0.785		0.795	−0.130		0.785	−0.113	
2 Hostile	1.74	1.081	0.640	–	0.640		0.680	−0.013		0.637	−0.013	
4 Ashamed	1.91	1.119	0.776	–	0.775		0.762	−0.028		0.773	−0.010	
6 Nervous	2.48	1.265	0.576	–	0.575		0.579	*0.240*		0.595	*0.258*	
9 Afraid	1.99	1.172	0.778	–	0.778		0.752	−0.040		0.776	−0.018	
PA subscale									0.749			0.811
3 Alert	2.22	1.256	–	0.224	–	–	*0.554*	0.221		–		
5 Inspired	2.79	1.269	–	0.614	–	0.607	0.129	0.617		0.135	0.621	
7 Determined	3.05	1.236	–	0.810	–	0.800	0.045	0.808		0.045	0.805	
8 Attentive	3.43	1.169	–	0.791	–	0.797	−0.054	0.788		−0.049	0.788	
10 Active	3.49	1.273	–	0.678	–	0.688	−0.176	0.690		−0.171	0.687	

Note. I-PANAS-SF = International Positive and Negative Schedule Short Form; PA = Positive Affect; NA = Negative Affect

**Table 3:** Fit Indices for different Models

	Model	χ^2^	*df*	CFI	ΔCFI	TLI	RMSEA (90% CI)	Δ RMSEA	SRMR	Δ SRMR	AIC	BIC	ABIC
	10-CFA	2444.99	34	0.78	–	0.71	0.131 (0.127–0.136)	–	0.14	–	118,150.49	118,346.45	118,247.95
	9-CFA	1050.87	26	0.89	–	0.85	0.098 (0.093–0.103)	–	0.080	–	104,777.54	104,954.54	104,865.57
	10-ESEM	814.94	26	0.93	–	0.88	0.086 (0.081–0.091)	–	0.035	–	116,046.28	116,292.82	116,168.89
	9-ESEM	406.97	19	0.96	–	0.92	0.070 (0.065–0.077)	–	0.025	–	103,980.83	104,202.07	104,090.86
Gender	Female (*N* = 2104)	230.89	19	0.956	–	0.916	0.073 (0.065–0.081)	–	0.025	–	53,056.34	53,254.14	53,142.95
	Male (*N* = 2002)	206.38	19	0.959	–	0.923	0.070 (0.062–0.079)	–	0.025	–	50,736.80	50,932.86	50,821.66
	M1(configural model)	436.98	38	0.958	–	0.920	0.072 (0.066–0.078)	–	0.025	–	103,793.13	104,235.55	104,013.12
	M2 (metric invariance)	485.79	52	0.954	0.004	0.936	0.054 (0.037–0.070)	0.018	0.031	0.006	103,807.63	104,161.56	103,983.62
	M3 (scalar invariance)	523.70	59	0.951	0.003	0.940	0.062 (0.057–0.067)	0.008	0.031	0.000	103,822.40	104,132.09	103,976.39
	M4 (item uniqueness invariance)	536.85	68	0.950	0.001	0.947	0.058 (0.053–0.063)	0.004	0.034	0.003	103,825.62	104,107.43	103,980.32
Grade	Grade 7 (*N* = 866)	107.98	19	0.953	–	0.911	0.074 (0.060–0.087)	–	0.028	–	22,526.60	22,693.34	22,582.19
	Grade 8 (*N* = 1034)	107.99	19	0.962	–	0.927	0.067 (0.055–0.080)	–	0.026	–	26,606.33	26,779.27	26,668.11
	Grade 9 (*N* = 1204)	121.66	19	0.965	–	0.934	0.067 (0.056–0.079)	–	0.024	–	30,208.07	30,386.34	30,275.17
	Grade 10 (*N* = 587)	107.47	19	0.937	–	0.881	0.089 (0.073–0.106)	–	0.033	–	14,501.61	14,654.74	14,543.63
	Grade 11 (*N* = 420)	48.69	19	0.969	–	0.941	0.061 (0.040–0.082)	–	0.025	–	9886.98	10,028.39	9917.32
	M1(configural model)	488.48	95	0.958	–	0.921	0.071 (0.065–0.077)	–	0.027	–	103,729.60	104,835.84	104,279.77
	M2 (metric invariance)	608.65	151	0.951	0.007	0.942	0.061 (0.056–0.066)	0.010	0.040	0.013	103,746.46	104,498.71	104,120.58
	M3 (scalar invariance)	694.59	179	0.945	0.006	0.945	0.059 (0.055–0.064)	0.002	0.042	0.002	103,769.89	104,345.10	104,055.98
	M4 (item uniqueness invariance)	821.73	215	0.936	0.009	0.946	0.059 (0.054–0.063)	0.000	0.050	0.008	103,940.14	104,287.82	104,113.05

The 10-item two-factor ESEM solution demonstrated a marginal model fit to the data. The correlation between PA and NA was not significant (r = − 0.02, *p* = 0.08). Further examination of item loadings revealed that most items significantly loaded on their intended factors, although some items significantly cross-loaded on unintended factors. The cross-loading (*λ =* 0.55) of one item (“alert”) was much higher than its primary loading (*λ =* 0.22). This is consistent with the result of the 10-item CFA solution in which the item (“alert”) was problematic. After removing the item “alert”, the 9-item ESEM solution demonstrated a good and much improved model fit to the data (see Table 3). The standardized primary loadings ranged from 0.59 to .81 and cross-loadings ranged from − 0.17 to .25 with no cross-loadings being larger than their primary loadings (see Table 2). Therefore, we decided to remove the item “alert” from the final measurement model. The correlation between PA and NA was not significant (r = − 0.01, *p* = 0.65). The Cronbach’s alpha values of each subscale were found to be acceptable (Table 2).

### Nomological validity

Bivariate correlations were used to evaluate the nomological validity of the 9-item I-PANAS-SF subscales. PA was found to be positively associated with effort (r = 0.42, *p* < 0.01) and negatively associated with lack of concentration (r = − 0.19, p < 0.01) and worry (r = − 0.40, *p* = 0.05). NA was negatively associated with effort (r = − 0.21, p < 0.01) and positively associated with lack of concentration (r = 0.46, p < 0.01) and worry (r = 0.49, p < 0.01).

### Invariance analysis

The measurement invariance of the 9-item I-PANAS-SF ESEM model was examined by progressively adding invariance constraints across genders and grades. Table 3 presents the goodness-of-fit indices and information criteria for independent and invariance models. All models displayed a good fit to the data. Comparing the more constrained models with the less constrained models across genders and grades, no decreases in model fit (ΔCFI, ΔRMSEA, and ΔSRMR) exceeded the recommended cutoff values for the fit indexes. These results provide support for weak, strong and strict measurement invariance of the 10-item ESEM model across genders and grades. The invariance is further supported by the information criteria showing consistent decreases (BIC) or at least very low increases (AIC and ABIC) in the model comparisons.

## Discussion

We translated the I-PANAS-SF into Chinese and examined its factor structure and measurement invariance across gender and grades in a sample of Chinese adolescents in Hong Kong. It was found that the 9-item two-factor structure measurement model (ESEM) of the Chinese version of the I-PANAS-SF was supported and its measurement invariance was evidenced across gender and grades. The Chinese version of the I-PANAS-SF demonstrated satisfactory internal consistency reliability and reasonable nomological validity.

Although Thompson claimed that the purpose of the development of the I-PANAS-SF was to facilitate comparisons of PA and NA across cultures [13], recent research has suggested that participants from different cultures respond to some I-PANAS-SF items differently [18]. Thus, culture-specific measures of positive and negative affect are necessary.

In this study, we examined the two-factor structure of the Chinese version of the I-PANAS-SF using CFA and ESEM. One strength of the current study was the employment of the ESEM approach, which was strongly advocated in the exploration of the factor structure of instruments with uncertain relationships among factors (PA and NA in this study). The highly restrictive independent cluster model used in CFA studies has been criticized because in CFA, each item is allowed to load on one factor. The misspecification of zero-factor loadings usually leads to distorted factors with overestimated factor correlations that might lead to distortions in structural relations. Therefore, the usage of ESEM would provide a better understanding of the factor structure of the multidimensional scales, because it allows items to cross-load on other factors [28]. As expected, the findings of the current study suggest that the ESEM model was a better fit to the data than the CFA model was. PA was not associated with NA (− 0.01 to − 0.04), suggesting that the two factors are independent and distinctive. This result is inconsistent with most previous findings in Western populations but consistent with previous findings from Chinese university students (− 0.01 to 0.01) [37]. Although a Taiwanese population sample was included in the development stage of the I-PANAS-SF, the sample size was relatively small (*n* = 60) and details of the factor structure and inter-factor correlations were not reported [13]. To the best of our knowledge, this is the first study to examine the factor structure of the I-PANAS-SF in a representative Chinese population using both ESEM and CFA approaches. More replication studies are needed to further examine the factor structure of the instrument in this population.

The item “alert” was found to be problematic and possibly not applicable to Chinese adolescents in Hong Kong in this study. Similar results were reported in previous validation studies of Watson’s 20-item PANAS in Chinese university students [27, 37, 38], in which the item “alert” was removed. In both the PANAS and the I-PANAS-SF, alertness was characterized as a positive affective feeling. However, in Chinese culture, the meaning of “alert” emphasizes paying attention to stressful or changing situations and staying continuously prepared to respond to such situations, which is considered a negative feeling. This cultural difference results in respondents understanding the same term differently. The item “alert” was also found to be problematic in American children [11] and adolescents [26] and was removed from the PANAS. Therefore, in this study the item “alert” was removed from the PA subscale of the Chinese version of the I-PANAS-SF. Removal of the problematic item greatly improved the model fit to the data, with no cross-loadings being larger than their primary loadings.

Another strength of this study is that the measurement invariance of the factor structure of the Chinese version of I-PANAS-SF across genders and grades was examined, which was rarely reported in previous PANAS related studies in Chinese populations. Measurement invariance is one of the fundamental psychometric properties of psychometrically sound instruments. Our findings demonstrate that the factor structure of the Chinese version of the I-PANAS-SF was invariant across genders and grades in Chinese adolescents in Hong Kong. Specifically, weak, strong, and strict measurement invariance of the I-PANAS-SF across genders and grades were demonstrated. Weak invariance examines whether the change in each item score corresponds to the change in the factor score across groups. It was found that the male and female participants, as well as participants from different grades, interpreted the I-PANAS-SF items in a similar way. Strong invariance measures whether the values of the observed variables reflect the values of the latent variables the same way across different groups. In this study, the mean scores of participants from different groups on the I-PANAS-SF subscales were comparable. Finally, strict invariance examines whether meaningful and unbiased comparisons can be made across groups. Evidence of strict invariance means that any difference between groups is a true difference rather than a measurement artifact. The results suggest that the scores derived from the 9-item Chinese version of the I-PANAS-SF are comparable across genders and grades. In other words, the 9-item Chinese version of the I-PANAS-SF is appropriate for both male and female secondary school students as well as students from different grades (7–11). This is the first study to provide evidence for the measurement invariance of the factor structure of PANAS measures in a Chinese population.

PANAS-C has been widely used for measuring PA and NA in children and adolescents [11, 12]. The original PANAS-C includes 27 items whereas the reduced version PANAS-C includes 10 items. Recent studies suggest that the 10-item PANAS-C fits the two-factor model better, with advantage of easy use and time-saving, especially when the time is limited and the test battery is long [12, 39]. The 10-item PANAS-C and the 10-item I-PANAS-F is comparable in length but different substantially in contents, in which no overlapping item in PA scale and only one same item (afraid) in NA scale. Therefore, questions are open for researchers to further explore to what extent the two measures are different from and associated with each other.

Although the present study provides initial psychometric evidence for the Chinese version of the I-PANAS-SF, several limitations should be noted. First, convenience sampling was used, and only students from government and government-aided secondary schools in Hong Kong were invited to participate in the study. The results may not be generalizable to students from private and international schools. Moreover, in this study, the participants were treated as healthy students, with no physical or mental illness screening for those special populations. Third, only student-level variance was considered; class-level effects were not examined. Forth, only nomological validity was examined in this study, other validity tests such as convergent validity and discriminant validity were not included. Fifth, longitudinal invariance was not examined in this study. Therefore, future studies are expected to shed light on the limitations abovementioned by taking the class effects into consideration and further examining other psychometric properties such as convergent validity, discriminant validity and longitudinal measurement invariance.

## Conclusions

The present study provides initial evidence for the factor structure and measurement invariance of the Chinese version of the I-PANAS-SF. The results demonstrate that the Chinese version of the I-PANAS-SF is a two-factor structure scale, with acceptable nomological validity and satisfactory internal consistency. Further, the measurement invariance results indicate that the Chinese version of the I-PANAS-SF could be used among students in different genders and grades. This feature of the instrument may ensure the accuracy of group comparisons in future studies.

## Data Availability

The datasets used and/or analyzed during the current study are available from the corresponding author on reasonable request.

## Abbreviations

ABIC: Adjusted Bayesian information criterion
AIC: Akaike information criterion
BIC: Bayesian information criterion
CFA: Confirmatory factor analysis
CFI: Comparative fit index
CI: Confidence interval
EFA: Exploratory factor analysis
ESEM: Exploratory structural equational modeling
IMI: Intrinsic Motivational Inventory
I-PANAS-SF: International Positive and Negative Affect Schedule Short Form
MI: Modification index
MLR: Robust maximum likelihood
NA: Negative affect
PA: Positive affect
PANAS: Positive Affect and Negative Affect Schedule
PE: Physical education
RMSEA: Root mean square error of approximation
SAS-2: Sport Anxiety Scale-2
SRMR: Standardized root mean square residual
TLI: Tucker-Lewis index
